# Ring finger protein 6 promotes breast cancer cell proliferation by stabilizing estrogen receptor alpha

**DOI:** 10.18632/oncotarget.15384

**Published:** 2017-02-16

**Authors:** Yuanying Zeng, Xin Xu, Siyu Wang, Zubin Zhang, Yan Liu, Kunkun Han, Biyin Cao, Xinliang Mao

**Affiliations:** ^1^ Jiangsu Key Laboratory for Translational Research and Therapeutics of Neuro-Psycho- Diseases, Department of Pharmacology, College of Pharmaceutical Sciences, Soochow University, Suzhou, Jiangsu, 215123, P. R. China; ^2^ Department of Oncology, Suzhou Municipal Hospital East Campus, Suzhou, 215100, P.R. China; ^3^ Suzhou Institute of Systems Medicine, Center of Systems Medicine, Chinese Academy of Medical Sciences, Suzhou, 215123, P. R. China; ^4^ Jiangsu Key Laboratory of Preventive and Translational Medicine for Geriatric Diseases, Soochow University, Suzhou, 215123, P. R. China; ^5^ Key Laboratory of Protein Modification and Degradation, School of Basic Medical Sciences, Affiliated Cancer Hospital & Institute of Guangzhou Medical University, Guangzhou 511436, P. R. China

**Keywords:** ring finger protein 6, breast cancer, ERα, Bcl-xL, doxorubicin

## Abstract

Ring finger protein 6 (RNF6) is a key oncogene in both prostate cancer and leukemia, but its role is elusive in breast cancer. In the present study, we found that RNF6 was overexpressed in more than 70% of breast cancer tissues and it was associated with overall survival. RNF6 increased breast cancer cell proliferation, migration and reduced cell sensitivity to doxorubicin. Further studies showed that RNF6 was closely associated with increased expression of estrogen receptor, a critical factor in the development of breast cancers. RNF6 was found to induce ERα expression and increased its stability. In doxorubicin-resistant breast cancer cells, RNF6 was found to be elevated in association with increased ERα and anti-apoptotic Bcl-xL, but not pro-apoptotic Bim-1. In consistence with this finding, overexpression of ERα led to increased Bcl-xL but had no effects on Bim-1. Therefore, this study demonstrated that there exists an RNF6/ERα/Bcl-xL axle in breast cancer which promotes cancer cell proliferation and survival. Targeting the RNF6/ERα/Bcl-xL axle could be a promising strategy in the treatment of breast cancer.

## INTRODUCTION

Breast cancer is one of the most common forms of cancers in women. According to the information from World Cancer Report, It was estimated that there were 1.7 million new cases and 0.5 million cancer deaths in 2012 worldwide, which both ranked the highest among all female cancers [1]. The National Cancer Registry of China also reported that breast cancer has emerged as the most registered cancer in women in China. The average incidence of breast cancer in women has reached 42.55 per 100,000 and it increases with age [2]. The diagnosis and therapeutic advances have improved the survival of breast cancer patients with early stages, but some patients become resistant and refractory even with optimized therapeutic methods. Genetic complexity has been proposed as a key factor in breast cancer treatment and predictive outcomes.

The ring finger protein 6 (RNF6) is a ubiquitin ligase that was originally cloned in a genetic study of chromosomal rearrangements in myeloproliferative disorders [3]. Because of its mapping to chromosome 13q12 and mutations in human esophageal squamous cell cancers, RNF6 was originally believed as a tumor suppressor gene [4]. However, RNF6 was found to mediate an atypical ubiquitination of the androgen receptor (AR) and promotes the transcriptional activity and specificity of AR in prostate cancer [5]. Therefore, RNF6 is proposed as an oncogene in the development and progression of prostate cancers and it is required for prostate cancer growth [5]. Recently we found that RNF6 is overexpressed in hematological cancers, including leukemia, lymphomas and multiple myeloma as a direct gene of the transcription factor Pbx1 [6]. RNF6 induces the proliferation of leukemia cells, whereas knockdown of RNF6 delayed the growth of tumors derived from human leukemia cell line K562 in mice [6]. Therefore, RNF6 has been demonstrated as an oncogene, but its specific roles in breast cancers are not yet understood.

The present study found that RNF6 is overexpressed in more 70% of breast cancer tissues in comparison with individual para-cancerous tissues. RNF6 increases the stability of estrogen receptor alpha (ERα), a key player in the pathophysiology of breast cancers. Targeting the RNF6/ERα axle could be a promising strategy for the treatment of breast cancers.

## RESULTS

### RNF6 is overexpressed in various breast cancer tissues and breast cancer cell lines

**Figure 1:**
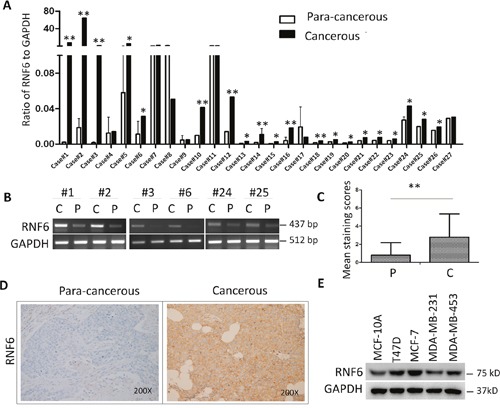
RNF6 is overexpressed in breast cancer **A**. Fresh primary breast cancer tissues and individual para-cancerous tissues were analyzed for RNF6 expression by qRT-PCR. **B**. RNF6 was detected by RT-PCR using tissues from representative patients as listed in A. C: Cancer tissues; P: para-cancerous tissues. **C**. Statistical analysis of human breast cancer tissue arrays (n=136) stained with an anti-RNF6 antibody. Immunostaining scores (mean ± SD) for RNF6 in para-cancerous (P) and cancerous (C) tissues were summarized. **D**. Representative fields of human breast cancer tissue arrays by immunohistochemical staining using an anti-RNF6 antibody. **E**. The whole-cell lysates from a normal breast cell line and various breast cancer cell lines were extracted, followed by detecting RNF6 protein levels measured by immunoblotting analyses. GAPDH was used as a loading control.*p<0.05, ** *p*<0.01.

RNF6 has been found as an oncogene that is highly expressed in prostate cancer and leukemia [5, 6]. To find out its expression profile in breast cancer, a panel of breast cancer tissues and their individual para-cancerous tissues were collected for RNF6 expression by qRT-PCR. As shown in Figure 1A, RNF6 was found to be upregulated in more than 70% (20/27) of representative primary breast cancer tissues compared with their para-cancerous control tissues. This finding was confirmed by RT-PCR as shown in Figure 1B. To further confirm the protein level of RNF6 in human breast cancer, 136 infitrating ductal carcinoma tissues were collected and tissue arrays were performed by immunohistochemical (IHC) staining with a specific RNF6 antibody. As shown in Figures 1C and 1D, RNF6 was highly expressed in human breast cancer compared with their para-cancerous tissues. All the results clearly showed that RNF6 was dysregulated in breast cancer tissues. To find out whether RNF6 was also expressed in breast cancer cell lines, MCF-7, MDA-231, MDA-453, and T47D were subjected to immunoblotting for RNF6 protein expression. As shown in Figure 1E, all cell lines examined displayed a high level of RNF6, in contrast, it was unable to be detected in a normal breast tissue cell line MCF-10A. This finding was consistent to previous reports that RNF6 was dysregulated in cancers such as leukemia [6] and prostate cancers [5]. Of interest, among these cell lines tested, T47D and MCF-7 are highly expressing estrogen receptor and MDA-MB-231 and MDA-MB-453 express relatively low levels of ER [7]. These results probably implicated that RNF6 was associated with ER expression.

### RNF6 predicts a poor prognosis of breast cancer patients

**Figure 2:**
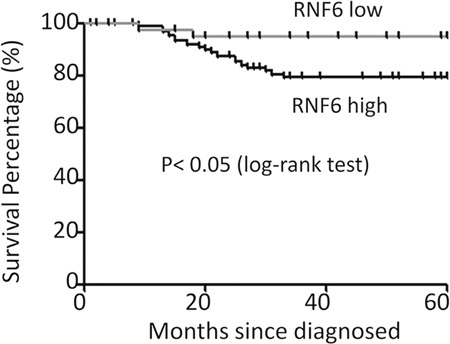
RNF6 is a negative index for the survival of breast cancer patients The survival periods of breast cancer patients were estimated using the Kaplan-Meier estimates as described previously (see Methods). All patients were classified into two groups based on the RNF6 expression level. Estimated survival percentage of each group of patients was calculated.

RNF6 was highly expressed in both breast cancer tissues and cell lines, we wondered whether RNF6 was clinically important. To this end, we next evaluated the association of RNF6 expression and the survival period of breast cancer patients. Patients were divided into two groups based on RNF6 expression, RNF6-low (n=43) and RNF6-high (n=93). Survival rates of patients within 5 years (60 months) were calculated and compared using the Kaplan–Meier estimate [10]. The result indicated that RNF6 was associated with the survival period of breast cancer patients. As shown in Figure 2, > 95% of breast cancer patients with a low expression of RNF6 survived more than 60 months, while <80% patients expressing a high level of RNF6 survived over 60 months. This analysis suggested that RNF6 probably predicted a poor prognosis of breast cancer patients.

### RNF6 is associated with age, clinical stage, ER and PR expression in patients with invasive ductal breast carcinoma

The above studies had showed that RNF6 was highly expressed in breast cancer tissues and was associated with patient survival, to further measure the effects of RNF6 in primary patients, 136 breast cancer tissues were applied for clinical evaluation in terms of patients' age, clinical stage, the expression of RNF6, ERα, PR and HER2, because the trio of ERα, PR and HER2 are proposed indicators for the prognosis of breast cancer patients. The *Chi* square analyses revealed that RNF6 expression was significantly associated with age. Patients > 50 years old expressed a higher level of RNF6 than younger ones. In addition, RNF6 was associated with clinical stages and the expression of ERα and PR but not HER2 (Table 1). Compared with patients with negative ERα and/or PR expression, a high level of RNF6 was detected in patients with positive ERα (*p* = 0.018) and PR (*p* = 0.002, Table 1). Therefore, we hypothesized that RNF6 was probably associated with the expression of ERα and PR.

**Table 1:** RNF6 is associated with patient age, clinical stage, ER and PR expression in breast cancer tissues

Variables	RNF6 Staining			*P* value*
	High	Low	Total	
**Age (years)**				
<50	25	19	44	0.045
≥50	68	24	92	
**Lymph node metastasis**				
Negative	64	31	95	0.502
Positive	30	11	41	
**Histology grade**				
1	0	1	1	0.402
2	42	18	60	
3	51	24	76	
**Clinical stage**				
I-II	62	47	109	0.019
III-IV	22	5	27	
**ER**				
Negative	9	12	21	0.018
Positive	80	35	115	
**PR**				
Negative	7	13	20	0.002
Positive	82	34	116	
**HER2**				
Negative	19	12	31	0.237
Positive	76	29	105	

^*^P value was calculated by using the *Chi* square (χ^2^) analysis based on RNF6 expression levels and patient numbers in each group.

### RNF6 promotes proliferation and migration of breast cancer cells

**Figure 3:**
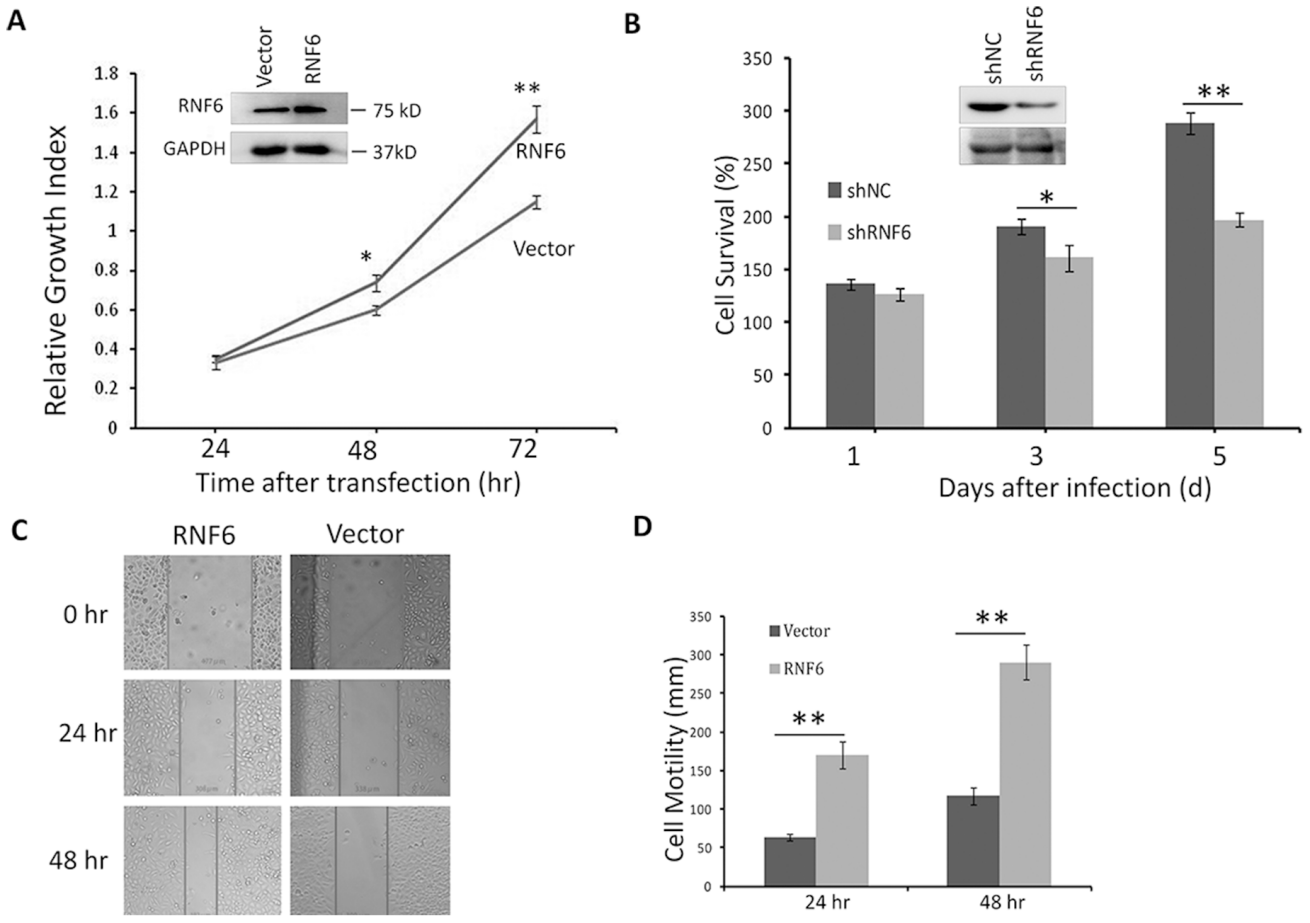
RNF6 promotes breast cancer cell proliferation in MCF-7 cells **A**. MCF-7 cells were transfected with a RNF6 plasmid or empty vector. Cells were then plated into 96-well plates and continued to culture for 24, 48 or 72 hrs before cell number counting. **B**. MCF-7 cells were infected with lentiviral shRNA of RNF6 (shRNF6) or negative control (shNC). Cell viability was evaluated at Day 1,3, and 5, respectively. **C** and **D**. RNF6-transfected or control MCF-7 cells were scratched with a tip, followed by culture in a 37°C incubator for 24 or 48 hrs. The wound gaps were analyzed by a microscope (C) followed by statistical analysis (D).

RNF6 was highly expressed in both breast cancer tissues and it was associated with poor prognosis, then we wondered whether RNF6 contributed to breast cancer cell proliferation. To this end, RNF6 plasmid was transfected into MCF-7 cells, followed by cell proliferation assay using MTT assay. The result showed that RNF6 promoted MCF-7 proliferation in a time-dependent manner (Figure 3A). Because RNF6 was overexpressed in MCF-7 cells, we next knocked down RNF6 in these cells by lentiviral shRNA (shRNF6) followed by cell proliferation assay. As shown in Figure 3B, RNF6 was markedly downregulated by shRNF6 which attenuated breast cancer cell proliferation.

Increased cell migration is critical for the malignancy of breast cancer, therefore, we next evaluated whether RNF6 contributed to such a feature in breast cancer cells. Using scratch wound healing assay, a widely accepted method to measure cell migration, we found that MCF-7 cells with transfected RNF6 displayed stronger healing ability than control cells (Figures 3C and 3D), suggesting that RNF6 might contribute to breast cancer cell migration.

### RNF6 increases breast cancer cell resistance to anti-cancer agents

**Figure 4:**
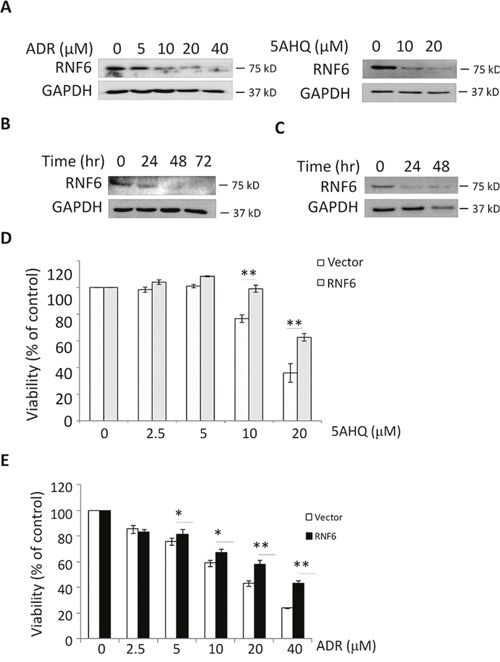
RNF6 increases MCF-7 resistance to anti-cancer agents **A**. MCF-7 cells were treated with increasing concentrations of doxorubicin (ADR) or A5HQ for 24 hrs, followed by cell lysate preparation and immunoblotting against RNF6 or GAPDH. **B** and **C**. MCF-7 was treated with ADR (16 M) or 5AHQ (20 μM) for indicated time points, followed by cell lysate preparation and immunoblotting against RNF6 or GAPDH. **D** and **E**. MCF-7 cells transfected with RNF6 plasmid or empty vector (as shown in Figure 1A) for 24 hrs, followed by treatment with 5AHQ (D) or ADR (E) at indicated concentrations for 48 hrs. Cell viability was measured by MTT.

Chemoresistance is an obstacle to clinical scientists and oncologists in breast cancer treatment. Previous studies showed that RNF6 has been found to be associated with chemoresistance of prostate cancer [5], therefore we wondered whether RNF6 also contributed to drug insensitivity of breast cancer. To this end, MCF-7 cells were treated with doxorubicin (ADR) [8], a mainstay drug in breast cancer treatment, or 5-amino-8-hydroquinoline (5AHQ) [9], a potential anti-cancer agent, for 24 hrs, followed by immunoblotting. The results showed that both ADR and 5AHQ could downregulate RNF6 expression in breast cancer cells in a concentration- and time-dependent manner (Figure 4A-4C). When transduced with a RNF6 plasmid, MCF-7 cells became resistant to 5AHQ (Figure 4D) and ADR (Figure 4E). This finding was consistent with the above study that RNF6 promoted breast cancer cell proliferation, migration and chemoresistance.

### RNF6 upregulates the expression level of ERα

**Figure 5:**
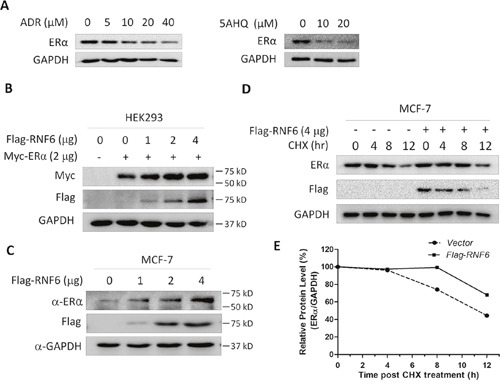
RNF6 increases the protein level of ERα **A**. MCF-7 cells were treated with increased ADR or 5AHQ for 24 hrs, followed by cell lysate preparation and immunoblotting assay with specific antibodies as indicated. These blots were stripped from Figure 4A and then subjected to analysis for ERα and GAPDH. **B**. RNF6 and ERα were co-tranfected into HEK293 cells. Forty-eight hours later, cells were lysed for immunoblotting analysis. **C**. MCF-7 cells were transfected with increased RNF6 plasmids, followed by immunoblotting against ERα, Flag and GAPDH. **D**. MCF-7 cells were transfected with Flag-RNF6 or vector for 24 hrs, followed by CHX chase assay. Immunoblotting analysis was performed against ERα and Flag. GAPDH was used as a loading control. **E**. Statistically analysis of Figure D.

The above histochemical tissue array studies suggested that RNF6 was associated with ERα, an important gene in breast cancer pathophysiology. Because RNF6 could be downregulated by anti-agents, we wondered whether the agents could also decrease ER expression. To this end, the same blots from Figure 4A in which MCF-7 cells treated with ADR and 5AHQ, respectively, were stripped and subjected to immunoblotting against ERα. As shown in Figure 5A, ERα was downregulated by both agents, in a similar manner to the effects of RNF6 (Figure 4A). This results implicated that RNF6 probably modulates ERα expression.

Previous studies have demonstrated that RNF6 as a ubiquitin ligase stabilized AR protein in prostate cancer patients [5], therefore, we evaluated the effects of RNF6 on the protein expression of ERα. As shown in Figure 5B, RNF6 increased ERα in a concentration-dependent manner in HEK293 cells when co-transfection of RNF6 and ERα. We next wondered whether RNF6 had any effects on the protein level of endogenous ERα. To this end, MCF-7 cells were transfected with RNF6 followed by immunoblotting. As shown in Figure 5C, the endogenous expression level of ERα was also markedly induced by RNF6. The above findings were confirmed by measuring ERα stability in the presence of RNF6 after protein synthesis was prevented by cycloheximide (CHX). As shown in Figure 5D and 5E, RNF6 markedly prevented degradation of ERα and significantly extended the half life of ERα.

### RNF6 stabilizes the ERα/Bcl-xL axle in a ubiquitination-independent manner

**Figure 6:**
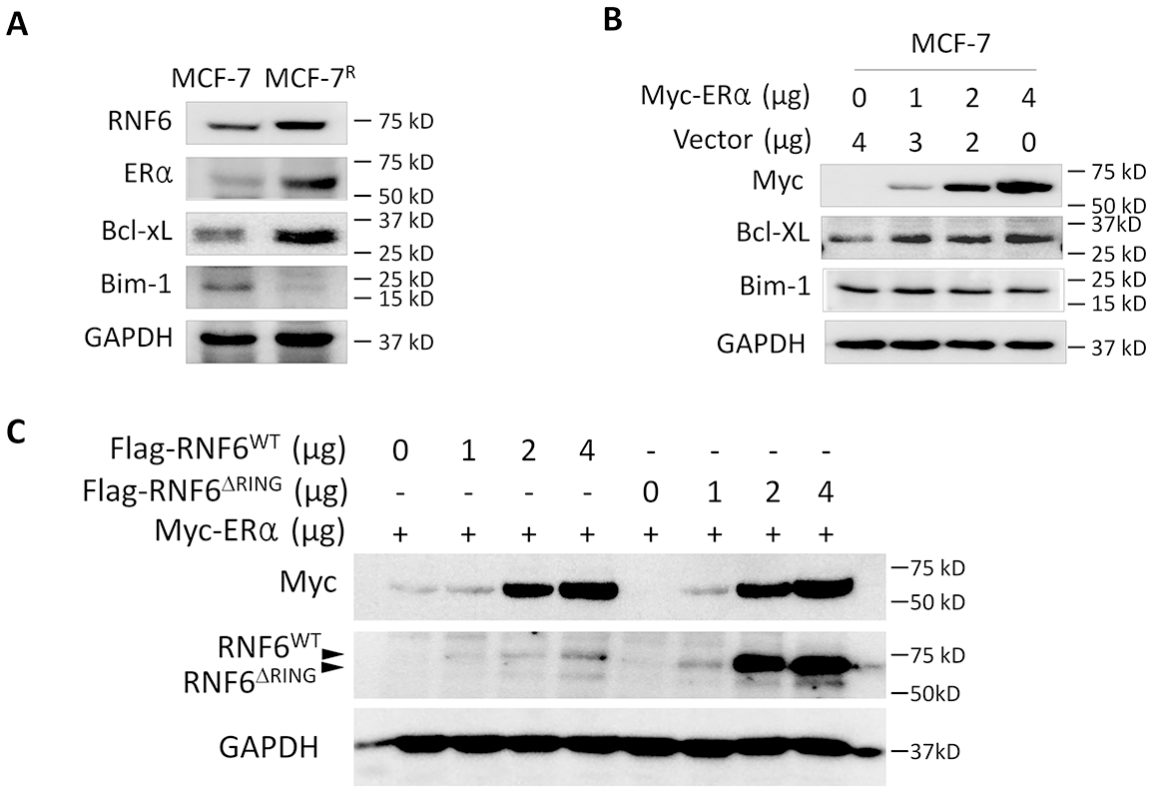
RNF6 promotes the ERα/Bcl-xL axle in breast cancer **A**. ADR-resistant MCF-7 (MCF-7^R^) cells were measured associated gene expression. **B**. MCF-7 cells were transfected with increased Myc-ERα plasmids for 48 hrs, followed by immunoblotting analysis. **C**. Wild-type or RING-deficient RNF6 plasmids were co-transfected with Myc-ERα in HEK293 cells. Forty-eight hours later, cells were harvest for immunoblotting analysis against Myc and Flag. GAPDH was used as a loading control.

The above studies showed that RNF6 stablizes ERα, to find out its significance in breast cancer, we analyzed several genes in ADR-resistant MCF-7 (MCF-7^R^) cells. As shown in Figure 6A, both RNF6 and ERα were raised in MCF-7^R^ cells compared with the wild-type parental MCF-7. Because MCF-7^R^ is resistant to ADR [10], we wondered whether there were any changes of the pro-survival and pro-apoptotic proteins using Bcl-xL and Bim as a representative, respectively [11, 12]. As shown in Figure 6A, Bcl-xL was upregulated while Bim-1 was downregulated in MCF-7^R^ cells, which was consistent with previous reports and the sensitivity of MCF-7^R^ cells to ADR. We subsequently wondered whether ERα could modulate the expression of Bcl-xL and Bim-1. To this end, MCF-7 cells were transfected with an ERα plasmid followed by immunoblotting. As shown in Figure 6B, Bcl-xL was increased by ERα but Bim-1 was not affected. Therefore, it could be concluded that RNF6 probably modulates the ERα/Bcl-xL axle in breast cancer cells.

RNF6 has been reported to be a ubiquitin ligase that mediates atypical ubiquitination of adrogen receptor and contributes to prostate cancer progression [5]. To find out whether RNF6 also excutes in a similar manner to stabilize ERα, we made a mutant RNF6 construct (RNF6^ΔRING^) that lacked the RING domain which is critical for the ubiquitinating activity. After co-transfected with ERα/RNF6^ΔRING^ or ERα/RNF6^WT^ cells were subject to immunoblotting. As shown in Figure 6C, both the wild-type and RING-deleted mutant RNF6 could increase ERα protein which suggesting that RNF6 raised the ERα protein probably in a manner rather than protein ubiquitination because if RNF6 acted as a ubiquitin ligase, its mutant lacking the RING domain will not increase ERα.

## DISCUSSION

The above studies demonstrated that the ubiquitin ligase RNF6 was highly expressed in breast cancers and it probably promotes cell proliferation, migration and pro-survival of breast cancer cells by modulating the ERα/Bcl-xL axle in a ubiquitination-independent manner.

RNF6 is first reported as a tumor suppressor gene not only because RNF6 is mapped to chromosome 13q12 [3], a location harboring several other suppressor genes such as p53 and PTEN, but also because its mutations are found in the gene of RNF6 in esophageal squamous cell carcinoma [4]. However, our present study demonstrated that RNF6 is oncogenic not only because of its high expression frequency in breast cancer tissues and cells, but because of its ability to increase breast cancer cell proliferation, migration and chemoresistance. This finding is consistent with its role in prostate cancer and leukemia [5, 6]. RNF6 as an oncogene promotes prostate cancer progression [5]. Recently, we found that RNF6 is highly expressed in leukemia, myeloma, and other hematological malignancies and contributes to leukemia cell proliferation *in vitro* and *in vivo* [6]. The present study showed that RNF6 is dysregulated in more than 70% of breast cancer tissues and it is negatively associated with the survival period of breast cancer patients, which suggesting RNF6 is probably associated with its specific pathophysiological activity.

HER2, ER and PR are critical biomarkers in the classification of breast cancers, predicating clinical outcomes and reference to therapeutic strategies [13, 14]. Based on the expression of this trio, one of the group is identified as tri-negative breast cancer (TNBC) which represents around 15% of all breast cancers and is characterized by shorter overall survival [15]. Based on our study, RNF6 is found to be associated with increased expression levels of ER and PR but not HER2, therefore, compared with the TNBC, although the survival period in patients with RNF6^low^ is markedly longer than those with RNF6^high^, RNF6 and associated ER expression might predict a favorable outcome compared TNBC. However, the underlying mechanisms are not known and deserves further investigation.

RNF6 is involved in prostate cancer progression by mediating the K63-chain ubiquitination on androgen receptor thus modulating AR transcriptional activity and specificity [5]. Different from MDM2, another ubiquitin ligase, that mediates polyubiquitination and stability of AR, RNF6 seems to have no effects on AR stability [5]. Although both AR and ER are nuclear receptors, RNF6 can stabilize ERα protein which was clearly demonstrated by exogenous and endogenous ERα proteins in presence of RNF6. It is well known that the RING domain is critical for the ubiquitin ligase activity of RNF6 and the deletion of the RING domain will abolish the ubiquitinating ligase activity [5]. As shown in the AR study, RNF6^ΔRING^ fails to induce AR polyubiquitination, however, this mutant can increase ERα stability in a potency similar to the wild type (Figure 6). Actually, co-transfection of RNF6^ΔRING^ leads to a raised level of AR protein [5]. Therefore, RNF6 modulates ERα and increases its stability in a manner different from ubiquitination.

RNF6 plays a critical role in prostate cancer progression by regulating AR transcriptional activity as a transcription factor because knockdown of RNF6 abolishes dihydrotestosterone-induced and androgen-independent AR activation. Specifically, RNF6-mediated ubiquitination modification is required for AR modulated transcription of a subset cognate target genes including PSA and RLN1 [5]. In breast cancer cells, three signature genes including ER, PR and HER2 determine chemoresistance and prognosis of breast cancers [13]. In the present study, RNF6 is highly associated with ER and PR but not HER2, specifically RNF6 stabilizes ERα and upregulates the expression of Bcl-xL, a key gene in cancer cell survival. Therefore, RNF6 probably regulate the ERα/Bcl-xL axle for the pathophysiology of breast cancer in a manner different from its role in prostate cancer and leukemia.

Taken together, the present study demonstrates that RNF6 triggers the ERα/Bcl-xL axle thus promoting proliferation, migration and chemoresistance of breast cancer cells. This study adds a novel line of evidence in breast cancer progression and may help in understanding breast cancer therapeutics.

## MATERIALS AND METHODS

### Primary breast cancer tissues and adjacent tissues

Fresh breast cancer tissues and corresponding adjacent normal tissues were collected from 32 female patients between 2013-2014 from the Suzhou Municipal Hospital East Campus, Suzhou, China. Of these cancer tissues, 27 were infitrating ductal carcinomas and 5 were intra-ductal carcinomas based on pathological studies. These tissues were preserved in liquid nitrogen for further studies. In addition, 136 infitrating ductal carcinoma tissues embedded in paraffin from Breast Cancer Bank of Suzhou Municipal Hospital East Campus were subjected to tissue arrays by immunostaining. The patient characteristics are summarized in Table 1. This study was conducted in accordance with the Declaration of Helsinki, International Conference on Harmonization Good Clinical Practice, and nationally mandated ethical requirements. The study protocol was reviewed and approved by the ethics committee of Suzhou Municipal Hospital and the written informed consent was obtained from the human subjects.

### Cell lines

Breast cancer cell lines including MCF-7, MDA-231, T47D and MDA-453 were originally obtained from American Type Culture Collection (Manassas, VA); Doxorubicin-resistant MCF-7 (MCF-7^R^) cells [16] were provided by Dr. Zhiyuan Zhong, Soochow University; HEK293 cells were obtained from Dr. Aaron Schimmer (the University of Toronto, Canada). Breast cancer cells and HEK293 cells were maintained in RPMI-1640 and Dulbecco's high glucose modified Eagle's medium (DMEM) (Hyclone), respectively. All media were supplemented with 10% fetal calf serum (Biowest^®^, Nuaillé, France), 100 μg/ml penicillin, and 100 U/mL streptomycin.

### Reverse transcription-polymerase chain reaction (RT-PCR) and Quantitative real-time polymerase chain reaction (qRT-PCR)

To determine the mRNA levels of RNF6, RT-PCR and qRT-PCR was performed as described previously [6].

### Preparation of RNF6 lentivirus and viral transduction

The full-length RNF6 gene was amplified by PCR as described previously [6]. The lentivirus-delivered shRNAs against RNF6 (shRNF6) together with the negative control (shNC) were purchased from GeneChem Co, Ltd (Shanghai, China). Lentiviruses were prepared with a standard protocol as manufacturer's instructions as described previously [6].

### Cell growth and viability

MCF-7 cells infected with RNF6 or shRNF6 or scramble lentivirus were cultured for 0 to 5 days at a density of 2 × 10^4^ cells/well in a 24-well plate. Cell viability was evaluated by MTT assay as described previously [17].

### Survival curve analysis

To estimate the survival period of breast cancer patients in association with RNF6, 136 patients were divided into two groups according to the expression level of RNF6 and the survival rate was calculated according to the Kaplan-Meier estimates described previously [18]. The Kaplan-Meier survival curve was plotted based on the survival rate of each group.

### Immunoblotting

Whole cell lysates were prepared as described previously [19]. Equal amounts (30 μg) of total proteins were subjected to sodium dodecylsulfate-polyacrylamide gel electrophoresis (SDS-PAGE) separation, followed by immunoblotting analyses with specific antibodies including antibodies against RNF6 (Thermo Fisher), Flag, Myc, HA (Medical & Biological Laboratories, Tokyo, Japan), ERα, Bcl-xL, Bim-1 (Cell Signaling Technologies., Ltd), or GAPDH (Abgent, Suzhou, China). Anti–mouse immunoglobulin G (IgG) and anti–rabbit IgG horseradish peroxidase conjugated antibody were purchased from R&D Systems.

### Immunohistochemistry

Breast cancer tissues were fixed in 10% neutral buffered formalin before being embedded in paraffin. The tissues were then cut into 6-micron thickness with a microtome. The slides were deparaffinized and rehydrated before antigen retrieval as described previously [20]. All slides were then subject to blocking in 10% normal serum for 10 minutes, followed by incubation with antibodies against RNF6, ERα, PR or HER2, overnight at 4°C. A biotin-conjugated secondary antibody diluted with Tris-based buffer (TBS) containing 10% serum and 1% BSA was applied to incubate for 10 minutes. After rinsing with cold TBS, the slides were then incubated with Streptavidin-peroxidase for 10 minutes before being stained with 3,3′-Diaminobenzidine (Beyotime, Nantong, China). The slides were finally stained with Hematoxylin and eosin before being mounted for microscopy analysis.

### Statistics

Values are expressed as means ± SD when necessary. The association of RNF6 expression and breast cancer tissues in patients samples were evaluated using the *Chi* square (χ^2^) analysis. The cell proliferation was evaluated with the student's *t* test. Survival time was calculated using the Kaplan–Meier method and compared by log-rank test as described previously [18].
